# Heart Failure with Supranormal Ejection Fraction: An Emerging High-Risk Phenotype Within the Preserved Ejection Fraction Spectrum—A Systematic Review

**DOI:** 10.3390/jcm15145361

**Published:** 2026-07-09

**Authors:** Andrea Sonaglioni, Giulio Francesco Gramaglia, Gian Luigi Nicolosi, Massimo Baravelli, Michele Lombardo, Italo Porto

**Affiliations:** 1 Division of Cardiology, IRCCS MultiMedica, 20123 Milan, Italy; massimo.baravelli@multimedica.it (M.B.); michele.lombardo@multimedica.it (M.L.); 2 Department of Emergency, Fondazione IRCSS Ca’ Granda, Ospedale Maggiore Policlinico, 20122 Milan, Italy; giulio.gramaglia@unimi.it; 3 Division of Cardiology, Policlinico San Giorgio, 33170 Pordenone, Italy; gianluigi.nicolosi@gmail.com; 4 Cardiovascular Disease Unit, IRCCS Ospedale Policlinico San Martino, 16132 Genoa, Italy; italo.porto@unige.it

**Keywords:** heart failure, supranormal ejection fraction, HFsnEF, heart failure with preserved ejection fraction, prognosis, ventricular remodeling, ventriculo-arterial coupling, higher ejection fraction

## Abstract

**Background**: Heart failure with supranormal ejection fraction (HFsnEF) has recently emerged as a distinct phenotype within the preserved ejection fraction spectrum. However, its clinical profile, underlying mechanisms, and prognostic significance remain incompletely understood. **Methods**: A systematic review was conducted according to PRISMA recommendations. PubMed, Scopus, and EMBASE were searched from inception to May 2026 for studies investigating HFsnEF. Demographic, clinical, laboratory, echocardiographic, prognostic, and therapeutic data were extracted. Weighted descriptive analyses were performed to compare HFsnEF and conventional heart failure with preserved ejection fraction (HFpEF) populations. Random-effects meta-analyses of study-specific event rates were conducted for all-cause mortality and for the composite endpoint of cardiovascular death or heart failure hospitalization. **Results**: Fourteen studies involving 17,158 HFpEF patients and 7493 HFsnEF patients were included. Compared with HFpEF, HFsnEF patients exhibited broadly similar clinical, laboratory, haemodynamic, and treatment profiles, with a higher prevalence of women and a lower prevalence of coronary artery disease representing the most consistent clinical differences. Conventional laboratory parameters and natriuretic peptide concentrations showed substantial overlap between groups. Echocardiographically, HFsnEF was characterized by significantly smaller left ventricular end-systolic dimensions and volumes and higher ejection fraction despite similar stroke volume and cardiac output. Mechanistic studies further demonstrated significantly higher end-systolic elastance and lower ventriculo-arterial coupling ratios despite comparable arterial elastance, supporting the presence of distinct ventricular–vascular mechanical properties. Prognostic analyses consistently identified HFsnEF as a phenotype associated with adverse clinical outcomes. Pooled event-rate analyses demonstrated significantly higher rates of both all-cause mortality and the composite endpoint of cardiovascular death or heart failure hospitalization in HFsnEF compared with HFpEF. Emerging evidence also suggests a potential benefit of sodium–glucose cotransporter-2 inhibitors in this population. **Conclusions**: HFsnEF appears to represent a distinct and potentially high-risk phenotype within the preserved ejection fraction spectrum. Its characteristic structural, haemodynamic, and prognostic features challenge the traditional assumption that progressively higher ejection fraction necessarily reflects better cardiovascular health. Further prospective studies incorporating advanced imaging and haemodynamic assessment are needed to refine its pathophysiological characterization and therapeutic management.

## 1. Introduction

Heart failure (HF) remains a major global health challenge, affecting millions of individuals worldwide and representing a leading cause of hospitalization, healthcare expenditure, and mortality [1]. Contemporary HF classification is largely based on left ventricular ejection fraction (LVEF), distinguishing heart failure with reduced ejection fraction (HFrEF), mildly reduced ejection fraction (HFmrEF), and preserved ejection fraction (HFpEF) [2,3]. This classification has important therapeutic implications because several evidence-based treatments have demonstrated substantial benefits in patients with reduced LVEF, whereas therapeutic responses appear more heterogeneous across the preserved ejection fraction spectrum [4,5,6].

Over the last decade, increasing evidence has challenged the traditional assumption that progressively higher LVEF values necessarily reflect better cardiac performance and prognosis. Epidemiological studies have consistently reported a U-shaped relationship between LVEF and adverse outcomes, with the lowest mortality observed within an intermediate range of preserved systolic function and a progressive increase in risk at both lower and higher LVEF values [7,8]. These observations have generated growing interest in patients exhibiting supranormal LVEF, generally defined by values exceeding 65%, and have led to the emergence of the concept of heart failure with supranormal ejection fraction (HFsnEF) [9,10].

Accumulating data suggest that HFsnEF may represent more than simply the upper extreme of the HFpEF spectrum. Compared with patients with conventional HFpEF, individuals with HFsnEF appear to exhibit distinctive clinical, structural, and haemodynamic characteristics, including female predominance, smaller left ventricular cavity dimensions, lower end-systolic volumes, higher end-systolic elastance, altered ventriculo-arterial coupling, increased diastolic stiffness, and impaired preload reserve [11]. These findings support the hypothesis that HFsnEF may constitute a specific pathophysiological phenotype characterized by a small, stiff, and hypercontractile ventricle rather than a truly “supernormal” cardiac function.

Particular attention has recently focused on the potential role of ventricular–vascular interaction in the development of HFsnEF [12]. Emerging invasive haemodynamic studies suggest that supranormal ejection fraction may be associated with increased left ventricular end-systolic elastance, altered ventriculo-arterial coupling, reduced ventricular capacitance, and impaired cardiovascular reserve despite apparently preserved systolic function at rest [13,14,15]. Such abnormalities may contribute to the paradox whereby patients with the highest ejection fraction values frequently experience persistent symptoms, exercise intolerance, and adverse clinical outcomes.

Interest in HFsnEF has further increased following reports suggesting worse clinical outcomes compared with patients with conventional HFpEF [16,17]. Several investigations have identified HFsnEF as an independent predictor of mortality and cardiovascular events, supporting the concept that supranormal ejection fraction does not necessarily reflect superior cardiovascular health. Moreover, recent studies have highlighted substantial phenotypic heterogeneity within HFsnEF itself, suggesting the existence of multiple clinical and biological subgroups with distinct prognostic trajectories.

At the same time, important uncertainties remain regarding the optimal definition of HFsnEF, its prevalence, underlying mechanisms, prognostic implications, and potential therapeutic management. Although emerging evidence suggests that sodium–glucose cotransporter-2 inhibitors may confer clinical benefits in this population, dedicated therapeutic data remain scarce [18]. In addition, most available data have been derived from echocardiographic studies, whereas comprehensive mechanistic characterization using invasive haemodynamics and advanced cardiovascular imaging remains limited.

Given the rapidly expanding but fragmented literature, a comprehensive synthesis of the available evidence is currently lacking. Therefore, the aim of the present systematic review was to summarize the epidemiological, clinical, laboratory, echocardiographic, haemodynamic, pathophysiological, and prognostic characteristics of HFsnEF, while also exploring emerging therapeutic evidence and identifying current knowledge gaps that may guide future research in this evolving field.

## 2. Materials and Methods

### 2.1. Review Design and Reporting Standards

This systematic review was performed following the Preferred Reporting Items for Systematic Reviews and Meta-Analyses (PRISMA) 2020 statement [19]. The completed PRISMA checklist is provided in the Supplementary Materials (Supplementary Material File S1).

The review protocol was prospectively submitted to the International Platform of Registered Systematic Review and Meta-analysis Protocols (INPLASY) (registration number: INPLASY202660056; registration date: 12 June 2026). The full protocol is available in the Supplementary Materials (Supplementary Material File S2).

### 2.2. Literature Search Strategy

A comprehensive literature search was independently performed by two investigators to identify studies evaluating the clinical characteristics, pathophysiological mechanisms, echocardiographic findings, prognostic implications, and therapeutic aspects of HFsnEF.

Electronic searches were conducted in PubMed, Scopus, and EMBASE from database inception to May 2026. The search strategy combined Medical Subject Headings (MeSH) terms and free-text keywords related to heart failure and supranormal left ventricular systolic function. The main search terms included combinations of “heart failure”, “HFpEF”, “preserved ejection fraction”, “higher ejection fraction”, “high ejection fraction”, “supranormal ejection fraction”, “supra-normal ejection fraction”, “supernormal ejection fraction”, “hyperdynamic ejection fraction”, “left ventricular ejection fraction”, “HFsnEF”, “prognosis”, “outcomes”, “cardiac remodeling”, “hemodynamics”, and “echocardiography”. No restrictions regarding language, publication year, or geographic region were applied.

Given the broad scope of the review, studies investigating clinical presentation, laboratory findings, cardiac structure and function, haemodynamic characteristics, prognostic outcomes, biomarker profiles, and therapeutic interventions in HFsnEF were considered potentially eligible for inclusion. Reference lists of all included articles and relevant review papers were also manually screened to identify additional studies not captured through the electronic search.

The study selection process was independently conducted by two reviewers. Any discrepancies regarding study eligibility were resolved through discussion and consensus, with consultation of a third reviewer when required.

### 2.3. Study Eligibility and Screening Process

Studies were considered eligible if they enrolled adult patients with HF and specifically investigated HFsnEF. Prospective and retrospective cohort studies, cross-sectional investigations, registry analyses, and other observational studies were eligible, provided they reported extractable clinical, imaging, haemodynamic, prognostic, biomarker, or therapeutic data.

To ensure consistency across studies, eligible investigations were required to provide a clear definition of HFsnEF based on LVEF values exceeding the conventional preserved ejection fraction range. Given the absence of a universally accepted definition and the progressive evolution of this concept in the literature, studies using echocardiographically derived LVEF cut-offs ranging from >58% to ≥70%, including sex-specific thresholds, were considered eligible. The lower boundary of >58% was selected to encompass the pioneering outcome-based investigation by Ohte et al. [20], which identified an optimal prognostic threshold of 57.2% through diagnostic performance analysis and subsequently adopted a ≥58% cutoff for patient stratification.

To improve methodological homogeneity and ensure consistent phenotypic characterization, only studies defining HFsnEF according to echocardiographically derived LVEF were included. Investigations relying exclusively on cardiac magnetic resonance or other imaging modalities for HFsnEF classification were excluded.

Studies comparing HFsnEF with HFpEF, HFmrEF, HFrEF, or control populations were eligible, as were investigations exclusively focused on HFsnEF cohorts.

Only original peer-reviewed articles were included. Editorials, narrative reviews, systematic reviews, meta-analyses, conference abstracts, expert opinions, guidelines, letters without original data, and case reports were excluded. Animal studies, preclinical investigations, and studies conducted exclusively in pediatric populations were also excluded. When multiple publications originated from overlapping study populations, the report providing the most comprehensive dataset or the longest follow-up was preferentially included. Any uncertainty regarding study eligibility was resolved through discussion and consensus among the reviewers.

### 2.4. Data Extraction and Evidence Collection

Following study selection, two investigators independently extracted data using a predefined standardized form developed specifically for this review. All extracted information was subsequently cross-checked, and discrepancies were resolved through consensus after re-examination of the original articles.

The following study-level characteristics were collected whenever available: first author, year of publication, country, study design, sample size, patient setting, duration of follow-up, and the definition adopted for HFsnEF.

Clinical and demographic variables included age, sex distribution, body mass index, cardiovascular risk factors, comorbidities, New York Heart Association (NYHA) functional class, previous HF hospitalizations, and medical therapies. Laboratory variables comprised renal function indices, natriuretic peptides, hematologic parameters, metabolic markers, and other biomarkers reported by the individual studies.

Cardiac structural and functional characteristics were systematically collected, including left ventricular dimensions and volumes, wall thickness measurements, left ventricular mass, left atrial size and volume, indices of diastolic function, right ventricular function, pulmonary pressure estimates, and parameters describing ventricular–arterial coupling (VAC) and haemodynamic performance when available. Left ventricular ejection fraction, stroke volume, cardiac output, and other haemodynamic variables were extracted as reported in the individual studies. Given the substantial heterogeneity in imaging protocols, measurement techniques (including conventional echocardiography, invasive haemodynamic assessment, and cardiac magnetic resonance in selected studies), and the variable availability of specific parameters across studies, no attempt was made to standardize or recalculate these measurements. Consequently, pooled descriptive estimates for different variables were often derived from different subsets of studies and should not be interpreted as mathematically comparable or interdependent.

Particular attention was devoted to variables potentially contributing to the characterization of the HFsnEF phenotype, including cardiac geometry, chamber remodeling, filling pressures, ventricular contractility, and invasive haemodynamic measurements. Information regarding clinical outcomes, prognostic predictors, phenotypic classifications, biomarker or proteomic analyses, and therapeutic interventions was additionally extracted whenever reported.

Because of the heterogeneity of study designs and reported outcomes, data extraction was primarily aimed at providing a comprehensive characterization of HFsnEF across epidemiological, clinical, imaging, pathophysiological, prognostic, and therapeutic domains.

### 2.5. Evaluation of Methodological Quality

The methodological quality of the included studies was independently assessed by two reviewers using the National Institutes of Health (NIH) Quality Assessment Tool for Observational Cohort and Cross-Sectional Studies [21]. This instrument evaluates key methodological domains, including study population definition, participant selection, exposure and outcome ascertainment, measurement reliability, control of confounding factors, statistical methodology, and completeness of reporting.

Each study was evaluated across 14 predefined domains according to NIH recommendations. Individual items were rated as “Yes”, “No”, “Cannot Determine”, “Not Reported”, or “Not Applicable”. Studies were subsequently categorized according to their overall methodological quality as Good, Fair, or Poor, taking into account both the number of fulfilled criteria and the potential impact of specific methodological limitations on study validity.

In addition to the NIH risk-of-bias assessment, the overall certainty of the available evidence was qualitatively appraised according to the principal GRADE domains (risk of bias, inconsistency, indirectness, imprecision, and publication bias) [22]. Given the predominantly observational nature of the included studies and the descriptive scope of this systematic review, this assessment was intended to provide an overall appraisal of evidence certainty rather than a formal outcome-specific GRADE rating.

Quality assessment was performed independently by both reviewers. Any disagreement regarding individual NIH quality domains, overall methodological quality classification, or the qualitative certainty-of-evidence assessment was resolved through discussion and consensus after re-evaluation of the original articles.

### 2.6. Data Synthesis and Statistical Methods

A qualitative synthesis was performed to summarize the available evidence regarding the epidemiology, clinical profile, laboratory findings, cardiac structure and function, prognostic implications, and therapeutic aspects of HFsnEF.

To facilitate comparison between HFpEF and HFsnEF populations across the included studies, pooled study-level descriptive estimates were calculated for demographic, clinical, laboratory, and echocardiographic variables. Continuous variables were summarized as weighted medians with weighted interquartile ranges (IQRs), using study sample size within each phenotype group as the weighting factor. Because these estimates were derived from aggregated study-level data rather than individual patient data, they were intended to provide a descriptive characterization of the included populations rather than formal comparative effect estimates. Accordingly, all associated *p*-values should be regarded as exploratory and interpreted with caution.

To provide an overall assessment of outcome burden, random-effects meta-analyses of study-specific event rates were performed for all-cause mortality and for the composite endpoint of cardiovascular death or heart failure hospitalization. Pooled event rates were calculated separately for HFpEF and HFsnEF cohorts. Differences between pooled event rates were subsequently evaluated using random-effects models, accounting for variability in study populations, follow-up duration, and endpoint definitions across the included investigations. To assess the robustness of these findings, a sensitivity analysis was performed by excluding the only study that defined HFsnEF using an outcome-derived LVEF threshold of >58% [20], rather than the more commonly adopted threshold of ≥60%. Event rates for all-cause mortality and the composite endpoint were then recalculated and compared with those obtained in the primary analysis.

Given the substantial clinical and methodological heterogeneity among studies, including differences in patient settings, HFsnEF definitions, inclusion criteria, outcome measures, and adjustment strategies, no quantitative pooling of hazard ratios or other prognostic effect estimates was performed. Furthermore, the relatively limited number of studies reporting comparable adjusted risk estimates precluded the generation of reliable pooled prognostic effect sizes. Instead, prognostic findings were narratively synthesized and summarized according to the direction and consistency of the reported associations.

All statistical analyses were performed using custom statistical routines implemented in Python (version 3.12.4; Python Software Foundation, Wilmington, DE, USA), including weighted descriptive summaries and random-effects analyses of study-specific event rates. Statistical significance was defined as a two-sided *p*-value < 0.05.

### 2.7. Use of Artificial Intelligence Tools

Artificial intelligence-assisted software was used solely to improve language quality and readability during manuscript preparation. Specifically, ChatGPT, based on GPT-5.5 (OpenAI, San Francisco, CA, USA), was employed to support grammatical revision, stylistic refinement, and enhancement of textual clarity.

Artificial intelligence tools were not involved in the conception or design of the study, literature screening, study selection, data extraction, quality assessment, statistical analyses, interpretation of results, or formulation of the scientific conclusions. All methodological decisions, data analyses, and final content were independently developed, critically reviewed, and approved by the authors, who assume full responsibility for the accuracy and integrity of the work.

## 3. Results

### 3.1. Study Identification and Selection

**Figure 1:**
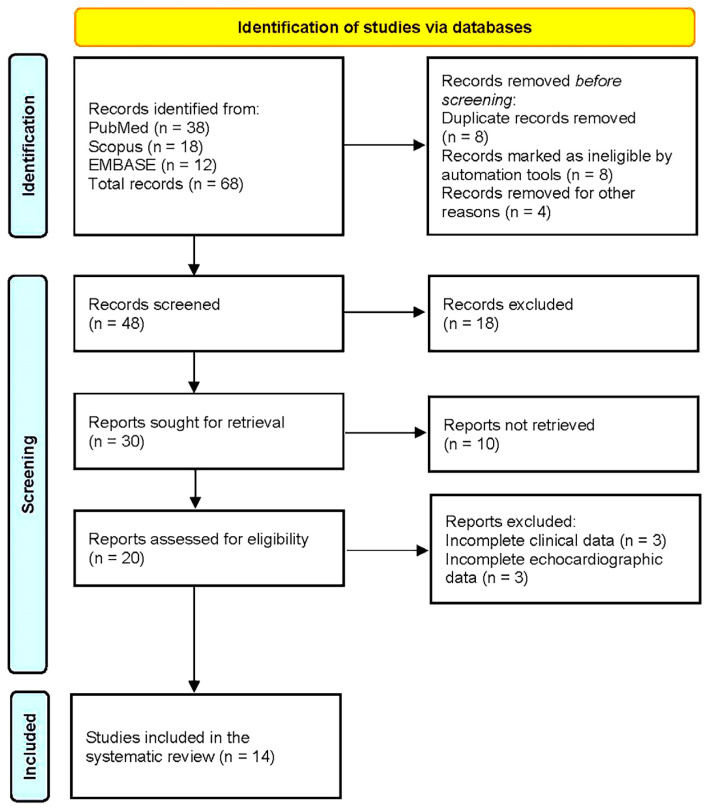
PRISMA flow diagram of study selection and eligibility assessment. Flowchart illustrating the identification, screening, eligibility evaluation, and final inclusion of studies in the systematic review according to the PRISMA 2020 framework. EMBASE, Excerpta Medica Database; PRISMA, Preferred Reporting Items for Systematic Reviews and Meta-Analyses.

The literature search yielded 68 records from PubMed, Scopus, and EMBASE. After removal of duplicate records, studies excluded by automated screening tools, and other ineligible records, 48 citations underwent title and abstract screening. Of these, 30 reports were considered potentially relevant and were retrieved for full-text assessment. Ten reports could not be retrieved despite attempts to obtain the complete manuscripts through institutional subscriptions, online databases, and alternative bibliographic sources, leaving 20 studies eligible for detailed evaluation. Following full-text review, six studies were excluded because of incomplete clinical and/or echocardiographic data. Ultimately, 14 studies published between 2022 and 2026 [13,14,15,20,23,24,25,26,27,28,29,30,31,32] fulfilled the predefined eligibility criteria and were included in the systematic review (Figure 1), providing data on 24,651 patients overall, including 17,158 with HFpEF and 7493 with HFsnEF.

### 3.2. Study Designs, Populations, and HFsnEF Definitions

The principal characteristics of the included studies are presented in Table 1.

Overall, the available literature included a heterogeneous mix of prospective and retrospective investigations, ranging from mechanistic haemodynamic studies and biomarker-based analyses to large-scale real-world registries. Study populations encompassed a broad spectrum of clinical settings, including hospitalized patients with acute or decompensated HF, ambulatory patients with chronic HF, elderly HFpEF cohorts, and individuals undergoing advanced invasive cardiovascular phenotyping. Sample sizes varied substantially, from small pathophysiological studies involving fewer than 50 participants to cohorts enrolling more than one thousand HFsnEF patients, thereby providing complementary insights into both disease mechanisms and clinical outcomes. Detailed evaluation of VAC and pressure–volume relationships was available in only a limited number of mechanistic studies.

Most investigations reported a predominance of women among HFsnEF patients, although the proportion of female participants varied considerably across studies. Geographically, the evidence originated primarily from Asia and Europe, with Japan contributing the largest number of studies. One additional mechanistic study originated from the United States and provided detailed invasive cardiopulmonary exercise testing and pressure–volume loop data in HFpEF patients across the preserved ejection fraction spectrum.

A degree of variability was observed in the definition of supranormal ejection fraction. Most studies adopted an LVEF threshold of ≥65% or >65%, whereas some investigations used lower cut-offs above 58–60%, and one study employed sex-specific thresholds. Despite these methodological differences, all studies focused on patients located at the upper extreme of the preserved ejection fraction spectrum, supporting the concept of HFsnEF as a potentially distinct clinical entity.

### 3.3. Clinical Profile of HFpEF and HFsnEF Cohorts

The pooled clinical characteristics of the HFpEF and HFsnEF populations are summarized in Table 2.

Overall, both groups exhibited a broadly comparable demographic and clinical profile.

Patients with HFsnEF showed a significantly higher proportion of women compared with HFpEF. Although HFsnEF patients tended to be older, age differences were not statistically significant. In contrast, coronary artery disease (CAD) was significantly less prevalent among HFsnEF patients.

Most traditional cardiovascular risk factors, including hypertension, diabetes mellitus, dyslipidaemia, smoking history, and obesity, were similarly represented in the two groups. Likewise, the prevalence of major comorbidities such as atrial fibrillation, chronic obstructive pulmonary disease, chronic kidney disease, previous stroke, malignancy, dementia, anaemia, and electrolyte abnormalities showed no significant differences between HFpEF and HFsnEF populations. Functional status and prior HF burden were also comparable, with similar distributions of NYHA functional class and previous HF hospitalizations.

Diagnostic scores specifically designed for HFpEF were available in only a limited number of studies and showed similar values across the two groups.

Medical therapy was also largely comparable between HFpEF and HFsnEF cohorts. The use of renin–angiotensin system inhibitors, beta-blockers, mineralocorticoid receptor antagonists, diuretics, sodium–glucose cotransporter-2 inhibitors, and antithrombotic therapies did not differ significantly between groups. Similarly, heart rate and blood pressure values were comparable between HFpEF and HFsnEF patients.

### 3.4. Biochemical and Biomarker Profiles Across the Ejection Fraction Spectrum

The laboratory findings observed in HFpEF and HFsnEF cohorts are reported in Table 3.

No statistically significant differences were identified across any of the evaluated laboratory parameters, including haematological, renal, metabolic, hepatic, inflammatory, and neurohormonal markers.

Indices of renal function, serum electrolyte concentrations, haemoglobin levels, and markers of nutritional status were comparable between groups. Similarly, no significant differences were observed for markers of glycaemic control, lipid metabolism, thyroid function, liver biochemistry, systemic inflammation, or uric acid metabolism.

Circulating natriuretic peptide concentrations, including both BNP and NT-proBNP, were also similar between HFpEF and HFsnEF populations. Likewise, serum creatinine and estimated glomerular filtration rate did not differ significantly between groups.

### 3.5. Cardiac Structural and Functional Remodeling in HFsnEF

Table 4 reports the echocardiographic characteristics of HFpEF and HFsnEF patients.

Left ventricular ejection fraction was available in 12 HFpEF and 14 HFsnEF studies, whereas stroke volume and cardiac output were reported in only 3 vs. 3 and 3 vs. 4 studies, respectively. Moreover, two mechanistic studies [13,14] complemented conventional imaging with invasive haemodynamic assessment, while Rosch et al. [13] also employed cardiac magnetic resonance for volumetric evaluation. Accordingly, pooled descriptive estimates for LVEF, stroke volume, and cardiac output reflect heterogeneous measurement methodologies and different subsets of studies rather than identical patient populations.

Several conventional echocardiographic parameters were comparable between groups; however, significant differences emerged for selected measures of left ventricular geometry, systolic chamber mechanics, and ventricular–arterial interaction.

HFsnEF patients exhibited significantly smaller left ventricular end-systolic dimensions and volumes compared with HFpEF patients. In contrast, interventricular septal thickness, posterior wall thickness, relative wall thickness, left ventricular mass index, left ventricular end-diastolic diameter, and left ventricular end-diastolic volume did not differ significantly between groups. Stroke volume and cardiac output were also comparable across the two phenotypes.

Consistent with the study definitions, LVEF was significantly higher in HFsnEF. Advanced haemodynamic parameters were available in a limited number of studies. While effective arterial elastance (Ea) was comparable between groups, HFsnEF patients exhibited significantly higher end-systolic elastance (Ees) and significantly lower VAC ratios (expressed as Ea/Ees).

Indices of diastolic function were broadly similar between HFpEF and HFsnEF. No significant differences were observed for E/e′ ratio, left atrial anteroposterior diameter, or left atrial volume index. The prevalence of more-than-mild mitral regurgitation was reported in only one HFpEF study and two HFsnEF studies, whereas moderate-to-severe mitral regurgitation was available in six and five studies, respectively. Despite the limited availability of these data, no significant differences in the prevalence of moderate-to-severe mitral regurgitation were observed between HFpEF and HFsnEF (weighted median 25.5% vs. 30.0%, *p* = 0.78). Likewise, no significant between-group differences emerged for moderate-to-severe aortic or tricuspid valve disease.

Right ventricular structure and function were also largely similar. Right ventricular end-diastolic diameter, tricuspid annular plane systolic excursion, and estimated pulmonary artery systolic pressure did not differ significantly between HFpEF and HFsnEF patients.

### 3.6. Clinical Outcomes and Prognostic Implications of HFsnEF

Table 5 summarizes the available prognostic evidence in patients with HFsnEF.

Follow-up duration ranged from 6.0 to 53.4 months, and the evaluated endpoints included all-cause mortality, cardiovascular mortality, HF hospitalization, composite cardiovascular outcomes, and all-cause rehospitalization.

Several studies reported an association between HFsnEF and an increased risk of adverse clinical outcomes, including all-cause mortality, cardiovascular death, and composite endpoints incorporating HF hospitalization. However, the magnitude of risk and the persistence of this association after multivariable adjustment varied across studies.

Among the identified predictors of adverse outcome, recurrent findings included higher LVEF values within the supranormal range, atrial fibrillation, elevated E/e′ ratio, advanced age, valvular heart disease, cardiac chamber enlargement, and impaired right ventricular (RV)–pulmonary arterial (PA) coupling. In addition, one study reported a lower risk of cardiovascular death or heart failure hospitalization among HFsnEF patients receiving sodium–glucose cotransporter-2 inhibitor therapy [32].

**Figure 2:**
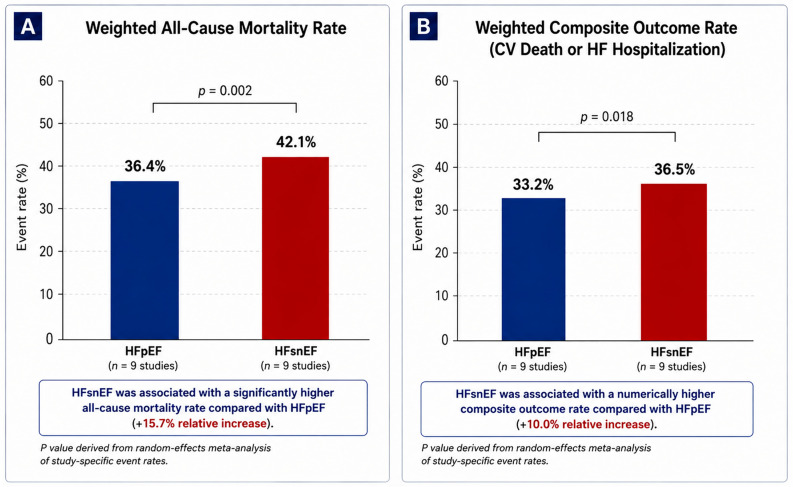
Comparative event rates for mortality and major clinical outcomes in HFpEF and HFsnEF. (**A**) Pooled all-cause mortality rates derived from random-effects meta-analysis of study-specific event rates in patients with HFpEF and HFsnEF. (**B**) Pooled rates of the composite endpoint of cardiovascular death or heart failure hospitalization according to heart failure phenotype. HFsnEF was associated with a significantly higher mortality burden and a greater incidence of composite adverse outcomes compared with conventional HFpEF. *p*-values were obtained from random-effects comparisons of pooled study-level event rates. CV, cardiovascular; HF, heart failure; HFpEF, heart failure with preserved ejection fraction; HFsnEF, heart failure with supranormal ejection fraction.

To provide an overall estimate of outcome burden, a random-effects meta-analysis of study-specific event rates was performed. HFsnEF was associated with a significantly higher weighted all-cause mortality rate compared with HFpEF. Similarly, the weighted rate of the composite endpoint of cardiovascular death or HF hospitalization was significantly higher in HFsnEF than in HFpEF. Figure 2 summarizes the pooled event rates derived from the available studies.

To assess the potential influence of the only study defining HFsnEF using an outcome-derived LVEF threshold of ≥58% [20], a sensitivity analysis was performed after excluding this investigation from the weighted event-rate analysis. The results remained essentially unchanged. The weighted all-cause mortality rate was 42.3% in HFsnEF versus 36.5% in HFpEF (relative increase: +15.7%), while the weighted composite endpoint rate (cardiovascular death or HF hospitalization) was 36.6% versus 33.3%, respectively (relative increase: +9.6%). These findings indicate that inclusion of the only study adopting an outcome-based ≥58% LVEF threshold did not materially influence the overall estimates or the main conclusions of the present systematic review.

### 3.7. Methodological Quality Assessment

The methodological quality assessment is shown in Table 6.

Overall, the included studies were characterized by a generally high methodological standard, supporting the reliability of the available evidence on HFsnEF. According to the NIH Quality Assessment Tool for Observational Cohort and Cross-Sectional Studies, 13 of the 14 included investigations were rated as *Good* quality, whereas one study was classified as *Fair* quality. Most studies clearly defined their research objectives, study populations, eligibility criteria, exposure measures, and outcome definitions. Follow-up duration was generally adequate for the assessment of clinical outcomes, and the majority of investigations employed appropriate statistical methodologies and multivariable adjustment strategies. Furthermore, outcome assessment procedures were consistently described across studies, reducing the likelihood of major ascertainment bias.

The most frequent methodological limitations were the absence of sample size justification or power calculations and the lack of repeated exposure assessments over time. As commonly encountered in observational HF research, blinding of outcome assessors was infrequently reported. In addition, several studies relied on retrospective data collection, which may have introduced residual confounding despite statistical adjustment. Nevertheless, the predominance of *Good*-quality studies provides a reasonably robust methodological foundation for the characterization of HFsnEF and its clinical implications.

The qualitative appraisal indicated an overall moderate level of certainty for the available evidence. This rating reflects the consistently good methodological quality of the included studies but was tempered by the observational design of all investigations, substantial clinical and methodological heterogeneity—including differences in patient populations, HFsnEF definitions, and outcome assessment—the relatively small sample size of several mechanistic studies, and the limited number of investigations contributing to specific analyses. Although no major concerns regarding indirectness were identified, publication bias could not be formally assessed because of the limited number of studies available for several outcomes. Collectively, these findings support HFsnEF as a distinct clinical phenotype while underscoring the need for larger prospective studies and randomized clinical trials to further strengthen the evidence base.

## 4. Discussion

### 4.1. Principal Insights from the Available Evidence

The present systematic review provides the most comprehensive synthesis to date of the available evidence on HFsnEF, integrating data from 14 studies involving 17,158 patients with HFpEF and 7493 patients with HFsnEF. The included investigations encompassed a broad range of clinical settings, including acute and chronic HF cohorts, nationwide registries, mechanistic haemodynamic studies, and biomarker-based analyses, thereby allowing a multidimensional characterization of this emerging phenotype.

Several important findings emerged from the present review. Despite substantial overlap in demographic characteristics, cardiovascular risk factors, comorbidities, laboratory findings, haemodynamic profile, and medical therapy, HFsnEF patients exhibited a significantly higher prevalence of female sex and a significantly lower prevalence of coronary artery disease compared with conventional HFpEF. In contrast, routine biochemical and biomarker profiles, including renal function indices, metabolic parameters, inflammatory markers, and natriuretic peptide concentrations, did not differ significantly between groups.

The most consistent differences between HFpEF and HFsnEF were observed at the level of cardiac structure and cardiovascular mechanics. Compared with HFpEF, patients with HFsnEF exhibited significantly smaller left ventricular end-systolic dimensions and volumes despite similar stroke volume and cardiac output. As expected, LVEF was substantially higher in HFsnEF. Importantly, studies evaluating ventricular–arterial interaction demonstrated comparable Ea but significantly higher Ees and lower VAC in HFsnEF. These findings suggest that the supranormal ejection fraction phenotype is primarily characterized by increased ventricular elastance and altered ventricular–vascular interaction rather than by differences in arterial load, providing additional evidence that HFsnEF possesses distinct cardiovascular mechanical properties beyond conventional ejection fraction assessment.

The available prognostic evidence consistently indicated that HFsnEF is associated with an increased burden of adverse clinical outcomes. In addition to multiple studies reporting an independent association between HFsnEF and worse prognosis, the present pooled analysis demonstrated significantly higher weighted rates of both all-cause mortality and the composite endpoint of cardiovascular death or HF hospitalization compared with conventional HFpEF. These findings further support the clinical relevance of HFsnEF and reinforce the need for dedicated risk stratification strategies within the preserved ejection fraction spectrum.

Taken together, the available evidence suggests that HFsnEF cannot be adequately described simply as the upper extreme of conventional HFpEF. Rather, it appears to represent a distinct phenotype characterized by specific structural, haemodynamic, and prognostic features that warrant dedicated mechanistic investigation and therapeutic evaluation.

### 4.2. Mechanistic Insights into the HFsnEF Phenotype

The paradox of HFsnEF lies in the observation that patients with the highest ejection fraction values often experience persistent symptoms and adverse clinical outcomes. Although conventional classification systems assume that progressively higher LVEF reflects better systolic performance, accumulating evidence suggests that excessively elevated ejection fraction may instead represent a marker of abnormal ventricular geometry, increased chamber stiffness, and altered ventricular–vascular interaction.

The structural and functional findings identified across the available studies support the concept that HFsnEF represents a distinct pathophysiological entity rather than simply the upper extreme of the HFpEF spectrum. Reduced ventricular capacitance and concentric remodeling may contribute to the development of apparently “supranormal” ejection fraction values despite the presence of impaired cardiovascular reserve. Because ejection fraction is intrinsically influenced by ventricular geometry, relatively small ventricular cavity dimensions may amplify LVEF values without necessarily reflecting superior cardiac performance [33].

Recent mechanistic investigations have further clarified the haemodynamic substrate underlying this phenotype. Rather than representing a state of enhanced systolic function, HFsnEF appears to be characterized by abnormalities in ventricular–vascular interaction and cardiovascular reserve, involving increased ventricular stiffness, altered chamber mechanics, and impaired VAC [34]. These alterations may become particularly relevant during exercise or physiological stress, when limited chamber expansion and reduced preload reserve can promote disproportionate increases in filling pressures, contributing to exertional intolerance and symptomatic HF despite preserved or supranormal resting systolic function [35]. This interpretation is consistent with invasive haemodynamic studies demonstrating that HFsnEF is characterized by a small, stiff ventricle with enhanced chamber contractile behaviour, a combination that may generate high ejection fraction values while simultaneously predisposing patients to adverse clinical manifestations and outcomes [36].

The predominance of elderly women among HFsnEF cohorts may also provide important mechanistic insights. Aging is associated with progressive stiffening of both the arterial tree and the left ventricle, a process that appears particularly pronounced in women and may contribute substantially to the pathophysiological substrate of HFsnEF [37,38,39]. With advancing age, central arteries undergo structural remodeling characterized by elastin fragmentation, collagen accumulation, medial thickening, and reduced vascular compliance, resulting in increased Ea and pulse pressure amplification [40]. Simultaneously, the left ventricle develops age-related concentric remodeling with increasing relative wall thickness, reduced chamber size, and enhanced Ees [41]. These ventricular and vascular alterations evolve in parallel and profoundly influence VAC [42].

Importantly, several population-based studies have demonstrated that women experience a steeper age-related increase in both arterial and ventricular stiffness than men, even in the absence of overt cardiovascular disease [37,43,44]. The disproportionate rise in ventricular Ees relative to Ea may lead to a haemodynamic state characterized by preserved stroke volume, reduced end-systolic volume, and apparently supranormal ejection fraction [45]. In this context, elevated LVEF may primarily reflect geometric and mechanical adaptations of a small ventricular chamber rather than truly enhanced myocardial performance. Reduced preload reserve may further accentuate this phenotype by limiting ventricular filling and promoting smaller chamber volumes despite preserved resting systolic function, particularly in elderly individuals, in whom age-related reductions in fluid intake and frequent diuretic use may contribute to relative intravascular volume depletion and diminished ventricular preload [46].

Age-related ventricular remodeling also contributes to progressive impairment of diastolic reserve [47]. Increased myocardial collagen content, extracellular matrix remodeling, cardiomyocyte hypertrophy, and impaired myocardial relaxation collectively reduce ventricular compliance and increase diastolic elastance [48]. Consequently, relatively small changes in preload may produce disproportionate elevations in filling pressures, especially during exercise, tachycardia, or volume loading [49]. This haemodynamic vulnerability may explain why many HFsnEF patients develop exertional dyspnoea and HF symptoms despite apparently preserved systolic function at rest.

The interaction between ventricular and vascular stiffening may represent a key pathophysiological mechanism linking HFsnEF with adverse outcomes. A stiff ventricle ejecting into a stiff arterial system becomes increasingly sensitive to changes in loading conditions, amplifying blood pressure fluctuations, myocardial wall stress, and filling pressure responses during physiological stress [50]. Furthermore, impaired VAC may reduce cardiovascular reserve and contribute to exercise intolerance despite preserved resting haemodynamics [35,51]. The observation that both all-cause mortality and the composite endpoint of cardiovascular death or HF hospitalization were significantly higher in HFsnEF than in HFpEF further supports the clinical relevance of these haemodynamic abnormalities. This framework is consistent with the growing concept that HFsnEF represents an advanced manifestation of age-related ventricular–vascular stiffening, particularly among elderly women.

Beyond left-sided ventricular–vascular interactions, growing evidence suggests that RV–PA uncoupling may represent an additional pathophysiological mechanism contributing to disease progression in HFsnEF. Chronic elevation of left ventricular filling pressures secondary to impaired diastolic compliance may be transmitted retrogradely to the pulmonary circulation, promoting pulmonary vascular remodeling, increased pulmonary arterial stiffness, and progressive elevation of right ventricular afterload [52]. Under these conditions, the right ventricle initially adapts through enhanced contractility; however, persistent pulmonary vascular loading may eventually exceed compensatory reserve, leading to RV–PA uncoupling and impaired right ventricular performance [53].

This mechanism may be particularly relevant in elderly HFsnEF patients, in whom longstanding ventricular stiffening and elevated filling pressures frequently coexist with pulmonary hypertension. Indeed, increasing evidence indicates that the prognostic impact of pulmonary hypertension in HF is strongly influenced by the ability of the right ventricle to adapt to increased afterload rather than by pulmonary artery pressure alone. Consequently, parameters integrating right ventricular systolic performance and pulmonary vascular load, such as the tricuspid annular plane systolic excursion to systolic pulmonary artery pressure (TAPSE/sPAP) ratio, have emerged as robust non-invasive markers of RV–PA coupling and adverse prognosis across the HF spectrum [54]. Furthermore, RV–PA uncoupling may contribute to exercise intolerance through multiple mechanisms, including reduced augmentation of right ventricular stroke volume during exertion, impaired pulmonary circulation reserve, reduced left ventricular preload during exercise, and worsening systemic venous congestion [55]. The coexistence of left ventricular diastolic dysfunction, ventricular–arterial stiffening, and RV–PA uncoupling may therefore identify a particularly advanced haemodynamic phenotype characterized by impaired biventricular reserve despite apparently preserved or supranormal resting ejection fraction. This integrated haemodynamic framework supports the concept that HFsnEF should not be viewed solely as a left-sided disorder but rather as a systemic cardiovascular syndrome involving complex interactions among ventricular stiffness, vascular dysfunction, pulmonary haemodynamics, and right ventricular adaptation.

**Figure 3:**
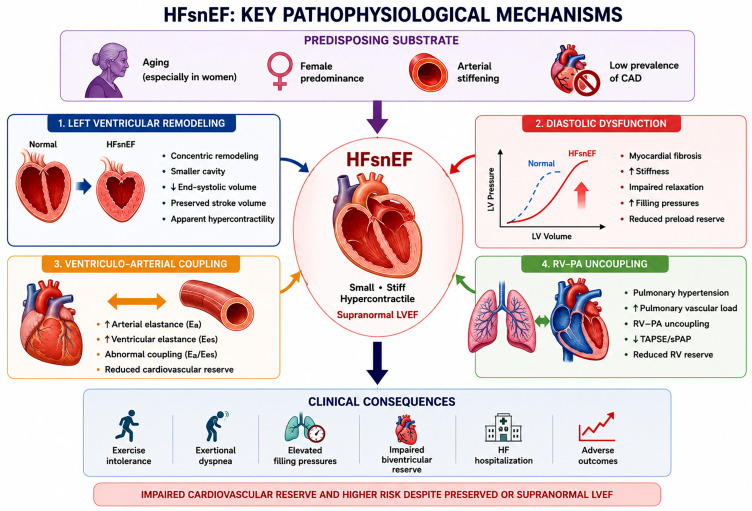
Proposed pathophysiological framework underlying heart failure with supranormal ejection fraction. Schematic representation of the principal mechanisms potentially contributing to the development and progression of HFsnEF. Aging, female predominance, increased arterial stiffness, and a lower prevalence of obstructive coronary artery disease may provide the biological substrate for this phenotype. Key pathophysiological features include concentric ventricular remodeling with reduced cavity size, impaired diastolic properties, abnormalities in ventriculo-arterial interaction characterized by increased ventricular and arterial elastance, and right ventricular–pulmonary arterial uncoupling. These alterations may collectively reduce cardiovascular reserve despite preserved or supranormal left ventricular ejection fraction, ultimately contributing to exercise intolerance, elevated filling pressures, heart failure hospitalization, and adverse clinical outcomes. CAD, coronary artery disease; Ea, effective arterial elastance; Ees, end-systolic elastance; HF, heart failure; HFsnEF, heart failure with supranormal ejection fraction; LVEF, left ventricular ejection fraction; RV–PA, right ventricular–pulmonary arterial; sPAP, systolic pulmonary artery pressure; TAPSE, tricuspid annular plane systolic excursion.

The principal pathophysiological mechanisms currently implicated in the development and progression of HFsnEF are schematically summarized in Figure 3, highlighting the complex interplay among age-related ventricular–vascular stiffening, concentric ventricular remodeling, diastolic dysfunction, abnormal VAC, RV–PA uncoupling, and impaired cardiovascular reserve.

Finally, the relatively low prevalence of CAD observed among HFsnEF patients represents an additional feature distinguishing this phenotype from other HF categories. While ischemic myocardial injury and adverse post-infarction remodeling are major drivers of disease progression in HFrEF and remain common among HFpEF populations, HFsnEF appears to be characterized predominantly by haemodynamic and structural alterations involving ventricular stiffness, impaired diastolic reserve, abnormal ventricular–vascular interaction, and progressive cardiovascular aging. Interestingly, the greater burden of CAD reported in HFpEF cohorts may partly reflect the increasingly recognized entity of heart failure with improved ejection fraction (HFimpEF) [56]. Indeed, a proportion of patients currently classified as HFpEF may have experienced previous left ventricular systolic dysfunction and subsequently recovered ventricular function following guideline-directed medical therapy and/or coronary revascularization, thereby contributing to the greater prevalence of CAD observed in some HFpEF cohorts [57].

### 4.3. Clinical Implications and Future Directions

The findings of the present review have several potentially important clinical implications. The available evidence indicates that supranormal ejection fraction should not be interpreted as synonymous with normal or superior cardiac function. Despite exhibiting a preserved or supranormal LVEF, patients with HFsnEF frequently demonstrate structural, haemodynamic, and prognostic characteristics that differ from those observed in conventional HFpEF. In particular, the significantly higher rates of both all-cause mortality and the composite endpoint of cardiovascular death or HF hospitalization observed in the present pooled analysis challenge the traditional assumption that progressively higher LVEF values necessarily reflect better cardiovascular health.

Recognition of HFsnEF as a potentially high-risk phenotype may improve risk stratification within the broad and heterogeneous HFpEF population [58,59]. Current HF classifications continue to group patients with ejection fractions ranging from 50% to values exceeding 70% within the same category, despite increasing evidence of important differences in ventricular geometry, cardiovascular mechanics, and clinical outcomes. Future diagnostic frameworks may therefore benefit from a more refined characterization of patients located at the upper extreme of the preserved ejection fraction spectrum.

The available therapeutic evidence remains limited. Although sodium–glucose cotransporter-2 inhibitors currently represent the most promising treatment strategy, dedicated randomized trials specifically designed for HFsnEF patients are lacking. The recent observation of a lower risk of cardiovascular death or HF hospitalization among HFsnEF patients receiving sodium–glucose cotransporter-2 inhibitors provides preliminary support for the potential applicability of contemporary HFpEF therapies to this phenotype [32]. However, whether treatment strategies should primarily target ventricular stiffness, abnormal VAC, impaired cardiovascular reserve, microvascular dysfunction, systemic inflammation, or extracardiac comorbidities remains uncertain.

From a practical perspective, greater attention should also be directed toward comprehensive haemodynamic assessment beyond conventional LVEF measurement. The present review identified significantly higher Ees and significantly altered VAC in HFsnEF compared with HFpEF, suggesting that these parameters may capture important aspects of cardiovascular mechanics not reflected by ejection fraction alone. VAC, commonly quantified by the Ea/Ees ratio [60,61], may therefore provide valuable insights into the interaction between ventricular performance and vascular load in patients with HFpEF and HFsnEF. Although only a limited number of studies have evaluated load-independent pressure–volume loop parameters across the preserved ejection fraction spectrum, the available evidence demonstrated substantial differences in ventricular stiffness, contractility, preload reserve, and ventricular–arterial interaction between patients with LVEF 50–60% and those with LVEF > 60%.

Similarly, assessment of RV–PA coupling, particularly through easily obtainable indices such as the TAPSE/sPAP ratio [62], may provide additional prognostic and pathophysiological information by identifying patients with impaired right ventricular adaptation to increased pulmonary vascular load. Given the growing evidence linking both abnormal ventriculo-arterial interaction and RV–PA uncoupling to exercise intolerance, elevated filling pressures, and adverse clinical outcomes, incorporation of these parameters into routine clinical evaluation may contribute to a more comprehensive characterization of patients with HFpEF and HFsnEF and facilitate the development of phenotype-oriented therapeutic strategies.

Future research should focus on improving phenotypic characterization through multimodality imaging, invasive haemodynamic assessment, biomarker profiling, and emerging proteomic approaches. Standardization of HFsnEF diagnostic thresholds and haemodynamic assessment protocols will also be essential to improve comparability across studies. Such strategies may help identify biologically distinct HFsnEF subgroups and facilitate the development of personalized therapeutic interventions. Given the substantial heterogeneity observed among currently available studies, a deeper understanding of the mechanisms underlying HFsnEF will be essential to establish whether this condition represents a truly independent HF phenotype or a final common manifestation of multiple pathophysiological pathways.

### 4.4. Strengths, Limitations, and Sources of Heterogeneity

The methodological rigor of the present review is supported by a comprehensive literature search, predefined eligibility criteria, independent study selection, standardized data extraction, and formal quality assessment. The combination of qualitative synthesis with pooled descriptive analyses and event-rate meta-analyses allowed a broad characterization of HFsnEF and its clinical outcome burden. In addition, the inclusion of studies spanning diverse clinical settings provided complementary insights into the structural, haemodynamic, pathophysiological, and prognostic features of this emerging phenotype.

Nevertheless, certain limitations should be considered when interpreting the findings. First, the overall evidence base remains relatively small and consists exclusively of observational studies, precluding definitive conclusions regarding causality. Second, substantial heterogeneity was present across studies with respect to patient populations, clinical settings, follow-up duration, endpoint definitions, and adjustment strategies. Third, no universally accepted definition of HFsnEF currently exists, with included studies adopting different ejection fraction thresholds and, in some cases, sex-specific criteria. Such variability may have contributed to differences in reported prevalence, clinical characteristics, and prognostic associations.

Other relevant limitations include the predominance of single-center investigations, the limited availability of invasive haemodynamic data, and the relatively small number of studies reporting detailed imaging, VAC, RV–PA coupling, and biomarker findings. Furthermore, the pooled descriptive analyses were based on study-level aggregated data and should therefore be considered exploratory. Likewise, the event-rate meta-analyses were not based on individual patient data and may have been influenced by differences in study design, follow-up duration, and outcome ascertainment.

An additional limitation relates to the heterogeneous assessment of left ventricular systolic function across the included studies. Although no significant between-group differences were observed in the prevalence of moderate-to-severe mitral regurgitation, the presence of relevant mitral regurgitation may influence the interpretation of volumetric indices because the difference between left ventricular end-diastolic and end-systolic volumes reflects the total stroke volume (forward stroke volume plus regurgitant volume) rather than the effective forward stroke volume. Consequently, LVEF and volumetrically derived stroke volume may overestimate true forward pump performance in patients with significant mitral regurgitation, particularly when Doppler-derived forward stroke volume is not systematically assessed. This limitation has been recognized as an important diagnostic challenge in patients with HFpEF and concomitant mitral regurgitation and should be considered when interpreting LVEF-based HF phenotypes [63].

An additional source of complexity relates to the inherent limitations of LVEF itself, which remains the cornerstone parameter used to define HF phenotypes. Although widely adopted in clinical practice, LVEF is influenced by loading conditions, geometric assumptions, image quality, endocardial border delineation, and operator expertise, and may demonstrate considerable inter-observer variability [64,65,66]. Moreover, LVEF has limited sensitivity for detecting early myocardial dysfunction and may remain within the normal or supranormal range despite the presence of significant abnormalities in ventricular stiffness, myocardial energetics, ventriculo-arterial interaction, cardiovascular reserve, or RV–PA coupling [67]. Emerging evidence also suggests that anthropometric factors, particularly chest wall conformation and thoracic geometry, may influence both the measurement and reproducibility of LVEF [68], further highlighting the limitations of relying exclusively on this parameter for HF classification and risk stratification.

## 5. Conclusions

HFsnEF appears to represent a distinct phenotype within the preserved ejection fraction spectrum rather than simply the upper extreme of conventional HFpEF. Compared with HFpEF, HFsnEF is characterized by a predominance of women, a lower prevalence of CAD, smaller left ventricular end-systolic dimensions and volumes, and distinct haemodynamic features, including higher Ees and lower VAC ratios (Ea/Ees), suggesting increased ventricular stiffness and contractile behavior despite similar arterial loading conditions.

Importantly, the available evidence challenges the traditional assumption that progressively higher ejection fraction necessarily reflects superior cardiovascular health. Despite exhibiting preserved or supranormal systolic function, patients with HFsnEF experienced significantly higher rates of all-cause mortality and cardiovascular death or HF hospitalization than patients with conventional HFpEF.

These findings support the concept of HFsnEF as a clinically relevant phenotype with unique structural, haemodynamic, and prognostic characteristics. Further prospective studies incorporating advanced imaging, invasive haemodynamic assessment, and standardized diagnostic criteria are needed to improve phenotypic characterization, risk stratification, and therapeutic management.

## Figures and Tables

**Table 1:** Characteristics of included studies investigating HFsnEF [13,14,15,20,23,24,25,26,27,28,29,30,31,32].

Study Name (Publication Year), Country	Study Design	Study Population	Sample Size (% Females)	Supranormal EF Cut-Off (%)
Rosch S. (2022), Germany [13]	Prospective, single-center	Symptomatic HFpEF patients (NYHA ≥ II) evaluated with invasive pressure–volume loop analysis	35 (80.0)	>60
van Essen B.J. (2023), Multinational [23]	Post hoc analysis of a prospective multicenter randomized clinical trial	Patients hospitalized for acute heart failure	155 (73.5)	>65
Ohte N. (2023), Japan [20]	Prospective, multicenter	Patients hospitalized for acute decompensated heart failure with LVEF > 40%	106 (61.3)	>58
Horiuchi Y. (2023), Japan [24]	Retrospective, single-center	Consecutive hospitalized heart failure patients	1943 (64.5)	>65
Popovic D. (2023), USA [14]	Retrospective, single-center	HFpEF patients undergoing invasive cardiopulmonary exercise testing and pressure–volume analysis	302 (65.0)	≥65
Ohte N. (2024), Japan [15]	Prospective, multicenter	Patients hospitalized for acute decompensated heart failure with LVEF > 40%	94 (64.9)	≥60
Huang L. (2024), China [25]	Retrospective, single-center	Hospitalized patients with heart failure and supranormal LVEF	221 (42.5)	≥65
Sonaglioni A. (2025), Italy [26]	Retrospective, single-center	Elderly HFpEF patients hospitalized with a first diagnosis of heart failure	101 (75.2)	≥65
Landucci L. (2025), Sweden [27]	Retrospective nationwide registry study	Patients with HFpEF enrolled in the Swedish Heart Failure Registry	1180 (52.0)	≥65
Stępień K. (2025), Poland [28]	Retrospective, single-center	Hospitalized patients with heart failure and LVEF ≥ 50%	40 (70.0)	>65
Segev A. (2025), Israel [29]	Retrospective, single-center	Hospitalized and ambulatory patients with heart failure and LVEF > 40%	1092 (68.4)	≥65
Sakata Y. (2025), Japan [30]	Prospective, multicenter	Chronic heart failure patients (Stage C/D) enrolled in a biomarker-proteomic study	47 (29.8)	Men > 70; Women > 75
Suzuki S. (2026), Japan [31]	Prospective, multicenter	Ambulatory patients with compensated chronic heart failure and LVEF > 40%	1423 (55.0)	>65
Inoue N. (2026), Japan [32]	Single-center target trial emulation study	HFsnEF patients with structural heart disease and elevated natriuretic peptides	562 (56.6)	≥65

This table summarizes the methodological features and population characteristics of the studies included in the systematic review. Information is provided on study design, geographic origin, patient population, sample size, sex distribution, and the left ventricular ejection fraction threshold used to identify HFsnEF. The included studies encompass mechanistic investigations, observational cohorts, registry analyses, and multicenter studies, offering a comprehensive overview of the currently available evidence on this emerging heart failure phenotype. HF, heart failure; HFpEF, heart failure with preserved ejection fraction; HFsnEF, heart failure with supranormal ejection fraction; LVEF, left ventricular ejection fraction; NYHA, New York Heart Association.

**Table 2:** Comparative clinical characteristics of patients with HFpEF and HFsnEF.

Clinical Parameter	Number of Studies (HFpEF vs. HFsnEF)	HFpEF Weighted Median (IQR)	HFsnEF Weighted Median (IQR)	Exploratory *p*-Value
Age (years)	12 vs. 14	77.0 (76.0–81.3)	79.6 (77.0–84.0)	0.66
Female sex (%)	12 vs. 14	51.5 (43.0–54.6)	64.5 (55.0–68.4)	**0.013**
BMI (kg/m^2^)	10 vs. 12	28.9 (22.6–30.2)	23.7 (22.5–29.9)	0.82
Hypertension (%)	12 vs. 14	76.0 (64.8–80.0)	74.5 (72.2–80.0)	1.00
Diabetes mellitus (%)	12 vs. 14	32.7 (27.0–37.8)	28.0 (19.0–37.5)	0.44
Dyslipidemia (%)	5 vs. 7	24.9 (24.9–28.2)	25.1 (24.7–47.5)	0.53
Smoking (%)	5 vs. 6	8.0 (8.0–12.0)	11.2 (7.0–14.0)	0.66
Coronary artery disease (%)	12 vs. 14	33.5 (29.6–41.0)	17.8 (14.2–27.6)	**0.009**
H2FPEF score	1 vs. 2	5.0	5.2 (4.8–6.0)	N/A
HFA-PEFF score	1 vs. 1	4.6	4.8	N/A
Atrial fibrillation (%)	12 vs. 14	49.0 (42.6–58.8)	43.0 (39.7–55.7)	0.33
COPD (%)	8 vs. 9	13.9 (8.1–23.2)	15.0 (7.5–21.9)	0.77
Prior stroke (%)	6 vs. 6	15.0 (8.1–18.2)	13.1 (7.7–18.8)	0.91
Cancer history/malignancy (%)	6 vs. 5	15.0 (9.1–15.4)	14.0 (10.0–19.8)	0.97
CKD (%)	5 vs. 5	38.9 (17.1–57.6)	25.0 (16.4–78.2)	0.86
Dementia (%)	2 vs. 2	19.0 (2.7–35.3)	20.3 (3.9–36.6)	1.00
Anemia (%)	4 vs. 4	22.1 (15.1–58.6)	22.7 (5.0–66.6)	0.94
Hyponatremia (%)	2 vs. 2	11.9 (8.6–15.1)	20.8 (5.0–27.7)	0.69
Hypernatremia (%)	1 vs. 1	10.1	20.8	N/A
NYHA class III (%)	8 vs. 9	40.5 (31.0–60.6)	39.0 (16.2–60.0)	0.87
NYHA class IV (%)	8 vs. 9	9.1 (0–31.0)	5.7 (0–60.4)	0.92
Previous HF hospitalization (%)	4 vs. 4	49.7 (22.0–68.2)	43.8 (16.0–68.9)	0.80
Heart rate (bpm)	12 vs. 14	75.0 (70.0–79.2)	75.0 (70.0–80.0)	0.91
Systolic BP (mmHg)	12 vs. 14	137.0 (129.0–147.0)	132.0 (125.1–142.8)	0.43
Diastolic BP (mmHg)	6 vs. 7	75.0 (71.0–77.1)	72.2 (66.1–80.0)	0.79
RASi/ARNi (%)	12 vs. 14	70.0 (63.9–80.0)	67.0 (49.6–71.7)	0.56
β-blockers (%)	12 vs. 14	74.5 (57.2–86.0)	68.0 (50.0–80.0)	0.59
Calcium-channel blockers (%)	7 vs. 8	43.0 (26.3–47.9)	42.5 (24.7–59.6)	0.81
MRAs (%)	10 vs. 12	40.0 (23.0–46.2)	33.6 (21.0–46.7)	0.58
Diuretics (%)	12 vs. 14	71.8 (34.0–87.1)	63.0 (23.8–87.3)	0.65
SGLT2 inhibitors (%)	4 vs. 5	13.7 (2.3–25.0)	21.0 (1.3–23.0)	0.83
Statins (%)	8 vs. 9	48.8 (32.3–65.2)	42.6 (29.7–67.5)	0.77
Digoxin (%)	7 vs. 8	8.1 (4.0–18.2)	7.5 (2.8–23.8)	0.94
Antiplatelets (%)	7 vs. 8	48.4 (22.0–56.7)	38.6 (23.0–56.3)	0.63
Anticoagulants (%)	7 vs. 8	51.3 (36.4–60.0)	40.4 (32.7–56.0)	0.71

Continuous and categorical variables are reported as weighted medians and interquartile ranges derived from aggregated study-level data. Reported *p*-values are exploratory and should be interpreted as descriptive comparisons rather than formal effect estimates. Significant exploratory *p*-values are shown in bold. AF, atrial fibrillation; ARNi, angiotensin receptor–neprilysin inhibitor; BMI, body mass index; BP, blood pressure; CAD, coronary artery disease; CKD, chronic kidney disease; COPD, chronic obstructive pulmonary disease; HF, heart failure; HFA-PEFF, Heart Failure Association Pre-test Assessment, Echocardiography and Functional Testing, Final Etiology score; HFpEF, heart failure with preserved ejection fraction; HFsnEF, heart failure with supranormal ejection fraction; H2FPEF, Heavy, Hypertensive, Atrial Fibrillation, Pulmonary Hypertension, Elder, Filling Pressure score; IQR, interquartile range; MRA, mineralocorticoid receptor antagonist; NYHA, New York Heart Association; RASi, renin–angiotensin system inhibitor; SGLT2, sodium–glucose cotransporter-2.

**Table 3:** Laboratory and biomarker profiles in HFpEF and HFsnEF populations.

Laboratory Parameter	Studies (HFpEF vs. HFsnEF)	HFpEF Weighted Median (IQR)	HFsnEF Weighted Median (IQR)	Exploratory *p*-Value
Hemoglobin (g/dL)	10 vs. 11	12.1 (11.0–12.7)	11.5 (10.9–13.2)	0.73
Hematocrit (%)	3 vs. 4	37.6 (37.0–38.1)	38.3 (37.6–39.0)	0.55
Albumin (g/dL)	5 vs. 6	3.60 (3.50–4.10)	3.68 (3.55–4.20)	0.72
Creatinine (mg/dL)	9 vs. 10	1.14 (1.00–1.24)	1.12 (1.00–1.35)	0.93
eGFR (mL/min/1.73 m^2^)	10 vs. 13	54.0 (49.0–58.0)	54.0 (44.5–62.0)	0.96
Sodium (mEq/L)	6 vs. 8	140.0 (139.4–140.5)	140.0 (139.0–140.1)	0.80
Potassium (mEq/L)	5 vs. 7	4.30 (4.10–4.40)	4.20 (4.00–4.40)	0.76
Total cholesterol (mg/dL)	3 vs. 3	182 (155–182)	185 (151–185)	0.88
LDL cholesterol (mg/dL)	4 vs. 4	89.0 (83.0–97.8)	93.0 (81.2–99.5)	0.80
HDL cholesterol (mg/dL)	4 vs. 4	46.0 (39.0–55.4)	53.6 (37.8–54.0)	0.62
Triglycerides (mg/dL)	3 vs. 3	123 (43–123)	122 (43–122)	0.94
AST (U/L)	5 vs. 5	24.4 (24.0–25.0)	24.3 (24.0–31.0)	0.87
ALT (U/L)	5 vs. 5	21.0 (21.0–21.1)	20.0 (19.0–22.9)	0.73
CRP (mg/L)	4 vs. 5	5.3 (0.2–7.0)	4.8 (2.0–9.8)	0.91
Glucose (mg/dL)	2 vs. 2	135.9 (108.1–135.9)	144.0 (115.3–144.0)	0.78
HbA1c (%)	3 vs. 4	6.0 (6.0–6.0)	6.1 (6.0–6.3)	0.83
Uric acid (mg/dL)	3 vs. 3	5.6 (5.6–7.3)	6.2 (6.2–8.5)	0.56
TSH (μIU/mL)	4 vs. 4	2.1 (1.49–2.9)	2.2 (2.0–2.91)	0.90
BNP (pg/mL)	4 vs. 4	229 (43.2–529)	305 (63.1–429)	0.75
NT-proBNP (pg/mL)	9 vs. 10	1250 (487–4363)	1178 (305–3583)	0.89

Data are presented as weighted medians and interquartile ranges calculated from aggregated study-level datasets. The table includes hematologic, renal, metabolic, hepatic, inflammatory, endocrine, and neurohormonal biomarkers reported across the included studies. Exploratory *p*-values are provided for descriptive comparison purposes and should not be interpreted as definitive measures of between-group differences. ALT, alanine aminotransferase; AST, aspartate aminotransferase; BNP, B-type natriuretic peptide; CRP, C-reactive protein; eGFR, estimated glomerular filtration rate; HbA1c, glycated hemoglobin; HDL, high-density lipoprotein; HFpEF, heart failure with preserved ejection fraction; HFsnEF, heart failure with supranormal ejection fraction; IQR, interquartile range; LDL, low-density lipoprotein; NT-proBNP, N-terminal pro-B-type natriuretic peptide; TSH, thyroid-stimulating hormone.

**Table 4:** Echocardiographic and Haemodynamic characteristics across the HFpEF–HFsnEF spectrum.

Echocardiographic Parameter	Number of Studies (HFpEF vs. HFsnEF)	HFpEF Weighted Median (IQR)	HFsnEF Weighted Median (IQR)	Exploratory *p*-Value
IVS (mm)	6 vs. 8	12.0 (9.8–13.3)	12.0 (10.0–14.0)	0.66
Posterior wall thickness (mm)	5 vs. 7	10.6 (9.5–11.0)	10.6 (10.0–12.0)	0.72
LVEDD (mm)	8 vs. 12	47.0 (44.2–48.0)	44.0 (42.5–49.0)	0.09
LVESD (mm)	6 vs. 9	31.0 (31.0–32.0)	27.0 (25.6–30.0)	**0.02**
Relative wall thickness	8 vs. 9	0.47 (0.40–0.50)	0.49 (0.40–0.51)	0.11
LV mass index (g/m^2^)	5 vs. 8	104.0 (98.4–119.5)	106.1 (101.0–112.0)	0.79
LVEDV (mL)	6 vs. 7	91.0 (65.7–128.0)	77.0 (57.4–112.0)	0.08
LVESV (mL)	6 vs. 7	40.5 (28.0–61.0)	27.0 (17.9–36.0)	**0.01**
Stroke volume (mL)	3 vs. 3	49.2 (48.6–69.0)	50.1 (49.8–72.0)	0.38
Cardiac output (L/min)	3 vs. 4	5.3 (3.4–5.3)	5.3 (3.3–6.4)	1.00
LVEF (%)	12 vs. 14	57.4 (55.0–63.8)	68.9 (65.0–74.3)	**<0.001**
GLS (%)	1 vs. 2	14.0	15.6 (15.6–15.6)	N/A
Ea (mmHg/mL)	3 vs. 3	1.80 (1.64–2.56)	2.00 (1.75–2.50)	0.72
Ees (mmHg/mL)	3 vs. 3	3.10 (1.33–5.90)	5.20 (1.85–6.15)	**0.04**
VAC (Ea/Ees)	3 vs. 3	0.82 (0.30–1.25)	0.48 (0.32–0.99)	**0.04**
E/e′	8 vs. 8	16.3 (14.1–17.4)	15.4 (12.4–18.0)	0.90
RVEDD (mm)	2 vs. 3	32.0 (32.0–32.0)	29.5 (22.0–29.5)	0.14
LA A-P diameter (mm)	3 vs. 4	45.9 (45.0–47.0)	45.0 (44.0–47.0)	0.83
LAVi (mL/m^2^)	6 vs. 6	46.2 (35.4–53.4)	46.5 (33.3–60.9)	0.95
More than mild MR (%)	1 vs. 2	2.0	77.9 (30.0–77.9)	N/A
Moderate-to-severe MR (%)	6 vs. 5	25.5 (14.9–45.4)	30.0 (16.6–34.0)	0.78
Moderate-to-severe AR (%)	4 vs. 3	25.5 (12.1–25.5)	19.9 (7.9–34.0)	0.87
Moderate-to-severe AS (%)	4 vs. 3	25.5 (10.1–25.5)	23.4 (14.8–34.0)	0.90
Moderate-to-severe TR (%)	5 vs. 4	25.5 (22.7–32.3)	31.7 (20.5–34.0)	0.73
TAPSE (mm)	4 vs. 4	19.0 (17.0–26.4)	19.0 (17.8–22.2)	0.67
sPAP (mmHg)	6 vs. 6	42.5 (30.0–53.0)	41.1 (35.0–49.7)	0.87

Variables include measures of left and right ventricular geometry, chamber volumes, systolic and diastolic function, valvular disease burden, pulmonary pressures, and indices of ventricular–vascular interaction. Values are reported as weighted medians and interquartile ranges derived from aggregated study-level data. Exploratory *p*-values are presented to facilitate descriptive comparisons between phenotypes and should be interpreted cautiously. Variables derived from fewer than three studies in either group are presented for descriptive purposes only and should likewise be interpreted with caution. Significant exploratory *p*-values are shown in bold. AR, aortic regurgitation; AS, aortic stenosis; Ea, effective arterial elastance; Ees, end-systolic elastance; GLS, global longitudinal strain; HFpEF, heart failure with preserved ejection fraction; HFsnEF, heart failure with supranormal ejection fraction; IVS, interventricular septal thickness; IQR, interquartile range; LA, left atrial; LAVi, left atrial volume index; LVEDD, left ventricular end-diastolic diameter; LVEDV, left ventricular end-diastolic volume; LVEF, left ventricular ejection fraction; LVESD, left ventricular end-systolic diameter; LVESV, left ventricular end-systolic volume; MR, mitral regurgitation; RVEDD, right ventricular end-diastolic diameter; sPAP, systolic pulmonary artery pressure; TAPSE, tricuspid annular plane systolic excursion; TR, tricuspid regurgitation; VAC, ventriculo-arterial coupling.

**Table 5:** Prognostic studies [15,20,23,24,25,26,27,28,29,30,32] and determinants of adverse outcomes in patients with HFsnEF.

Study Name	HFsnEF Sample Size	Follow-Up (Months)	Endpoint	Main Predictors of Adverse Events (OR/HR)
van Essen B.J. (2023) [23]	155	6.0	180-day all-cause death, CV death, non-CV death, HF/renal failure rehospitalization	HFsnEF associated with higher all-cause mortality (HR 1.45) and non-CV mortality (HR 2.65), but lower HF/renal failure rehospitalization risk (HR 0.61)
Ohte N. (2023) [20]	106	17.4	Composite of all-cause death and HF readmission	LVEF (HR 1.046), atrial fibrillation (HR 3.203), and E/e′ (HR 1.083) independently predicted outcome
Horiuchi Y. (2023) [24]	1943	29.0	Composite of cardiovascular death or HF readmission	HFsnEF associated with increased risk of the composite endpoint (HR 1.03) and all-cause mortality (HR 1.10) among women
Ohte N. (2024) [15]	94	17.4	Composite of all-cause death and HF readmission; HF readmission	Higher LVEF associated with worse event-free survival. TAPSE/sPAP independently predicted the primary endpoint, whereas sPAP alone did not
Huang L. (2024) [25]	221	53.4	Primary: all-cause mortality; Secondary: cardiovascular death and cardiovascular readmission	Valvular phenotype, cardiac chamber enlargement, and AF/AFL predominance characterized the subgroup with the highest mortality risk (HR 3.32)
Sonaglioni A. (2025) [26]	101	43.2	All-cause mortality or all-cause rehospitalization	Higher LVEF independently associated with adverse outcome (HR 1.04)
Landucci L. (2025) [27]	1180	25.0	All-cause mortality, CV mortality, non-CV mortality, HF hospitalization, all-cause hospitalization	Crude risk increased above LVEF ≈ 55%; association lost after multivariable adjustment
Stępień K. (2025) [28]	40	52.0	All-cause mortality	HFsnEF diagnosis (HR 1.665) and age (HR 1.037) independently predicted mortality
Segev A. (2025) [29]	1092	32.0	Primary: all-cause mortality; Secondary: total and HF-related hospitalizations	HFsnEF associated with increased mortality versus HFpEF in unadjusted (HR 1.258) and adjusted analyses (HR 1.112)
Sakata Y. (2025) [30]	47	49.2	Cardiovascular death or first HF hospitalization	HFsnEF associated with increased risk compared with HFnEF (HR 2.71)
Inoue N. (2026) [32]	562	12.3	Composite of cardiovascular death or HF hospitalization	SGLT2 inhibitor therapy associated with reduced risk (HR 0.63)

The table presents sample size, duration of follow-up, investigated endpoints, and the principal variables associated with adverse clinical outcomes. Reported prognostic measures include hazard ratios (HRs) or odds ratios (ORs), when available. The included studies provide insight into the relationship between HFsnEF, mortality risk, heart failure hospitalization, cardiovascular events, and factors influencing prognosis across different clinical settings. AF, atrial fibrillation; AFL, atrial flutter; CV, cardiovascular; E/e’, ratio between early transmitral flow velocity and early diastolic mitral annular velocity; HF, heart failure; HFpEF, heart failure with preserved ejection fraction; HFsnEF, heart failure with supranormal ejection fraction; HR, hazard ratio; LVEF, left ventricular ejection fraction; OR, odds ratio; sPAP, systolic pulmonary artery pressure; SGLT2, sodium–glucose cotransporter-2; TAPSE, tricuspid annular plane systolic excursion.

**Table 6:** Methodological quality assessment of included studies according to the NIH quality assessment tool for observational cohort and cross-sectional studies [13,14,15,20,23,24,25,26,27,28,29,30,31,32].

Study	Q1	Q2	Q3	Q4	Q5	Q6	Q7	Q8	Q9	Q10	Q11	Q12	Q13	Q14	Overall
Rosch S. (2022) [13]	Y	Y	NR	Y	NR	Y	NA	Y	Y	N	Y	NR	NA	Y	8 (Good)
van Essen B.J. (2023) [23]	Y	Y	Y	Y	NR	Y	Y	Y	Y	N	Y	NR	Y	Y	11 (Good)
Ohte N. (2023) [20]	Y	Y	NR	Y	NR	Y	Y	Y	Y	N	Y	NR	Y	Y	10 (Good)
Horiuchi Y. (2023) [24]	Y	Y	Y	Y	NR	Y	Y	Y	Y	N	Y	NR	Y	Y	11 (Good)
Popovic D. (2023) [14]	Y	Y	Y	Y	NR	Y	Y	Y	Y	N	Y	NR	Y	Y	11 (Good)
Ohte N. (2024) [15]	Y	Y	NR	Y	NR	Y	Y	Y	Y	N	Y	NR	Y	Y	10 (Good)
Huang L. (2024) [25]	Y	Y	NR	Y	NR	Y	Y	Y	Y	N	Y	NR	Y	Y	10 (Good)
Sonaglioni A. (2025) [26]	Y	Y	NR	Y	NR	Y	Y	Y	Y	N	Y	NR	Y	Y	10 (Good)
Landucci L. (2025) [27]	Y	Y	Y	Y	NR	Y	Y	Y	Y	N	Y	NR	Y	Y	11 (Good)
Stępień K. (2025) [28]	Y	Y	NR	Y	NR	Y	Y	Y	Y	N	Y	NR	Y	Y	10 (Good)
Segev A. (2025) [29]	Y	Y	NR	Y	NR	Y	Y	Y	Y	N	Y	NR	Y	Y	10 (Good)
Sakata Y. (2025) [30]	Y	Y	NR	Y	NR	Y	Y	Y	Y	N	Y	NR	Y	Y	10 (Good)
Suzuki S. (2026) [31]	Y	Y	Y	Y	NR	NA	NA	Y	Y	N	Y	NA	NA	N	7 (Fair)
Inoue N. (2026) [32]	Y	Y	NR	Y	NR	Y	Y	Y	Y	N	Y	NR	Y	Y	10 (Good)

Individual studies were evaluated across 14 predefined methodological domains addressing study design, participant selection, exposure and outcome assessment, control of confounding, follow-up adequacy, and statistical methodology. Overall study quality was classified as Good, Fair, or Poor according to NIH recommendations. This evaluation was used to determine the methodological robustness of the evidence included in the systematic review. NA, not applicable; NIH, National Institutes of Health; NR, not reported; Q1–Q14, methodological quality domains of the NIH Quality Assessment Tool; Y, yes.

## Data Availability

The dataset generated from the extraction of data from the included studies will be made openly accessible through Zenodo (https://zenodo.org; accessed 7 June 2026).

## Supplementary Materials

The following supporting information can be downloaded at https://www.mdpi.com/article/10.3390/jcm15145361/s1. File S1: PRISMA 2020 checklist; File S2: The full INPLASY record (https://inplasy.com/inplasy-2026-6-0056/, accessed on 12 June 2026).
