# Supplementary material for: Heart Failure with Supranormal Ejection Fraction: An Emerging High-Risk Phenotype Within the Preserved Ejection Fraction Spectrum—A Systematic Review
Source: J Clin Med. 2026 Jul 9;15(14):5361. doi: 10.3390/jcm15145361 (PMC13410013; doi:10.3390/jcm15145361)
Supplement: Supplementary file 1 [file jcm-15-05361-s001.zip › File S2.pdf]

International Platform of Registered Systematic Review and Meta-analysis Protocols

Sonaglioni et al. INPLASY protocol 202660056. doi:10.37766/inplasy2026.6.0056 Downloaded from https://inplasy.com/inplasy-2026-6-0056/

INPLASY

INPLASY202660056

doi: 10.37766/inplasy2026.6.0056

Received: 12 June 2026

Published: 12 June 2026

Corresponding author:
Andrea Sonaglioni

sonaglioniandrea@gmail.com

Author Affiliation:
MultiMedica.

Heart Failure with Supranormal Ejection Fraction: An Emerging High-Risk Phenotype Within the Preserved Ejection Fraction Spectrum—A Systematic Review

Sonaglioni, A; Gramaglia, GF; Nicolosi, GL; Baravelli, M; Lombardo, M.

ADMINISTRATIVE INFORMATION

Support - Ministero della Salute.

Review Stage at time of this submission - Completed but not published.

Conflicts of interest - None declared.

INPLASY registration number: INPLASY202660056

Amendments - This protocol was registered with the International Platform of Registered Systematic Review and Meta-Analysis Protocols (INPLASY) on 12 June 2026 and was last updated on 12 June 2026.

INTRODUCTION

Review question / Objective The purpose of this systematic review is to critically evaluate and synthesize the current evidence regarding heart failure with supranormal ejection fraction (HFsnEF), an increasingly recognized phenotype within the preserved ejection fraction spectrum. Specifically, the review aims to characterize its demographic profile, clinical features, comorbidities, laboratory findings, cardiac structural and functional characteristics, haemodynamic properties, and prognostic implications. Particular emphasis will be placed on identifying the pathophysiological mechanisms potentially underlying HFsnEF, including ventricular remodeling, ventricular–arterial interaction, and right ventricular–pulmonary arterial coupling. The review will also examine available evidence on clinical outcomes, including mortality and heart failure-related events, and summarize emerging data on therapeutic management. By integrating findings from observational studies, registries, mechanistic investigations, and outcome analyses, this review seeks to determine whether HFsnEF represents a distinct heart failure phenotype rather than simply the upper extreme of conventional HFpEF, while highlighting current knowledge gaps and priorities for future research.

Rationale Left ventricular ejection fraction (LVEF) remains the cornerstone of heart failure classification and therapeutic decision-making. While patients with preserved ejection fraction are traditionally considered a relatively homogeneous group, recent investigations have suggested that individuals with markedly elevated or supranormal LVEF may exhibit distinctive clinical and cardiovascular characteristics. Emerging evidence indicates that these patients often differ from conventional HFpEF populations with respect to ventricular geometry, myocardial mechanics, ventricular–vascular interaction, and clinical outcomes. Moreover, several studies have reported a paradoxical association between very high LVEF values and an increased risk of adverse cardiovascular events, challenging the long-held assumption that progressively higher ejection

fraction necessarily reflects superior cardiac performance and prognosis.

Despite growing interest in this topic, available data remain dispersed across studies with heterogeneous designs, populations, and definitions of HFsnEF. Consequently, a comprehensive synthesis of the evidence is lacking. A systematic review is therefore warranted to consolidate current knowledge, clarify the clinical and pathophysiological profile of HFsnEF, evaluate its prognostic significance, and identify areas requiring further investigation. Such information may contribute to a more refined understanding of heart failure phenotypes and support future research aimed at improving risk stratification and management strategies in this emerging population.

Condition being studied Heart failure with supranormal ejection fraction (HFsnEF), generally defined as heart failure occurring in patients with left ventricular ejection fraction values exceeding the conventional preserved ejection fraction range, represents an emerging and incompletely characterized cardiovascular phenotype. Although traditionally considered a marker of preserved or enhanced systolic performance, supranormal ejection fraction has recently been associated with unique structural and haemodynamic features, including smaller ventricular cavity size, altered ventricular–vascular interaction, increased ventricular stiffness, and impaired cardiovascular reserve. The present review will investigate the clinical characteristics, cardiac remodeling patterns, haemodynamic mechanisms, prognostic significance, and potential therapeutic implications of HFsnEF, with particular attention to its distinction from conventional heart failure with preserved ejection fraction (HFpEF). The review will also explore whether HFsnEF should be regarded as a separate heart failure phenotype within the preserved ejection fraction spectrum.

METHODS

Search strategy A systematic search of the literature will be conducted in PubMed/MEDLINE, Scopus, and EMBASE from database inception to May 2026. The search approach will combine controlled vocabulary terms and free-text keywords related to heart failure and supranormal left ventricular systolic function. Search terms will include, but will not be limited to, "heart failure", "preserved ejection fraction", "HFpEF", "supranormal ejection fraction", "supernormal ejection fraction", "higher ejection fraction", "hyperdynamic ejection fraction", "left ventricular ejection fraction", "HFsnEF", "cardiac remodeling", "ventricular–arterial coupling", "haemodynamics", and "clinical outcomes". No restrictions regarding language, publication date, or geographic location will be applied. In addition, the reference lists of eligible studies and relevant review articles will be manually screened to identify further potentially relevant publications not captured through the electronic database search. Study selection, eligibility assessment, and data extraction will be performed independently by two reviewers, with disagreements resolved through discussion and consensus.

Participant or population Adult patients diagnosed with heart failure and supranormal left ventricular ejection fraction (HFsnEF) will be eligible for inclusion. Studies enrolling individuals across a broad range of clinical settings, including ambulatory populations, hospitalized patients, acute or chronic heart failure cohorts, registry-based populations, and mechanistic haemodynamic studies, will be considered. Given the lack of a universally accepted definition, studies using echocardiographic left ventricular ejection fraction thresholds above the conventional preserved ejection fraction range (generally >60% to ≥70%, including sex-specific criteria when applicable) will be eligible. Studies comparing HFsnEF with heart failure with preserved ejection fraction (HFpEF), mildly reduced ejection fraction (HFmrEF), reduced ejection fraction (HFrEF), or non-heart failure control groups will be included when relevant. Only studies involving adult human participants will be considered, whereas pediatric populations, animal studies, and experimental preclinical investigations will be excluded.

Intervention No specific intervention will be required for study eligibility. The review will focus on patients with heart failure and supranormal left ventricular ejection fraction (HFsnEF) and will evaluate observational evidence describing their clinical characteristics, cardiac structure and function, haemodynamic profile, prognostic outcomes, biomarker patterns, and therapeutic management. Studies assessing pharmacological treatments, including sodium–glucose cotransporter-2 inhibitors and other heart failure therapies, will be considered when available; however, the primary objective of the review is phenotypic and prognostic characterization rather than the evaluation of a predefined therapeutic intervention.

Comparator Where available, patients with heart failure and supranormal ejection fraction (HFsnEF) will be compared with individuals across other

Sonaglioni et al. INPLASY protocol 202660056. doi:10.37766/inplasy2026.6.0056 Downloaded from https://inplasy.com/inplasy-2026-6-0056/

heart failure phenotypes, particularly heart failure with preserved ejection fraction (HFpEF). Additional comparator groups may include patients with mildly reduced ejection fraction (HFmrEF), reduced ejection fraction (HFrEF), or non-heart failure control populations, depending on the design of the individual studies. Studies exclusively evaluating HFsnEF cohorts without a comparison group will also be eligible for inclusion if they provide relevant information regarding clinical characteristics, cardiac structure and function, haemodynamic features, biomarkers, prognosis, or therapeutic management.

Study designs to be included Original observational studies investigating heart failure with supranormal ejection fraction (HFsnEF) will be eligible for inclusion. Accepted study designs will comprise prospective and retrospective cohort studies, observational registry analyses, cross-sectional studies, case–control studies, and mechanistic investigations incorporating advanced imaging or invasive haemodynamic assessment. Both single-center and multicenter studies will be considered. Post-hoc analyses of clinical trials may also be included when they provide original data specifically related to HFsnEF. Only peer-reviewed.

Eligibility criteria Studies will be considered eligible if they include adult patients with heart failure and specifically evaluate individuals with supranormal left ventricular ejection fraction (HFsnEF), defined according to echocardiographic ejection fraction values exceeding the conventional preserved ejection fraction range. Given the absence of a universally accepted definition, studies adopting thresholds above 60%, including higher or sex-specific cut-offs, will be eligible. Investigations reporting data on clinical characteristics, comorbidities, laboratory findings, cardiac structure and function, haemodynamic parameters, biomarkers, therapeutic management, or clinical outcomes will be considered for inclusion. Both comparative studies and studies exclusively focused on HFsnEF populations will be eligible.

Eligible study designs will include prospective and retrospective cohort studies, registry-based analyses, cross-sectional investigations, case–control studies, and mechanistic observational studies. Only full-text articles published in peer-reviewed journals and involving human adults will be included.

Studies will be excluded if they are reviews, meta-analyses, editorials, letters without original data, conference abstracts, expert opinions, case reports, animal studies, preclinical investigations, or studies conducted exclusively in pediatric populations. When multiple publications report data from overlapping cohorts, the study providing the most complete dataset or the longest follow-up will be retained.

Information sources The evidence base will be identified through systematic searches of the following electronic bibliographic databases: PubMed/MEDLINE, Scopus, and EMBASE. Searches will cover the period from database inception to May 2026. To maximize study identification, the reference lists of all included articles and relevant review papers will also be manually examined for additional eligible publications. No restrictions related to language, year of publication, or country of origin will be applied. Study selection and verification of eligibility will be conducted independently by two reviewers, with any disagreements resolved through discussion and consensus.

Main outcome(s) The primary outcomes of interest will be the clinical, structural, functional, haemodynamic, and prognostic characteristics associated with heart failure with supranormal ejection fraction (HFsnEF). Particular emphasis will be placed on differences in demographic profile, cardiovascular risk factors, comorbidities, laboratory findings, cardiac chamber geometry, ventricular systolic and diastolic function, ventricular–vascular interaction, and right ventricular–pulmonary arterial coupling. Clinical outcome measures, including all-cause mortality, cardiovascular mortality, heart failure hospitalization, and composite cardiovascular endpoints, will also be evaluated whenever reported. In addition, the review will examine prognostic factors associated with adverse outcomes and summarize available evidence regarding therapeutic strategies and their potential impact on clinical prognosis in patients with HFsnEF.

Additional outcome(s) Secondary outcomes will include the prevalence and distribution of specific echocardiographic and haemodynamic abnormalities associated with HFsnEF, such as changes in left ventricular dimensions and volumes, left atrial remodeling, filling pressure indices, ventricular stiffness, end-systolic elastance, effective arterial elastance, ventriculo–arterial coupling, right ventricular function, pulmonary pressure estimates, and right ventricular–pulmonary arterial coupling parameters. Additional outcomes will encompass biomarker and proteomic profiles, phenotypic

Sonaglioni et al. INPLASY protocol 202660056. doi:10.37766/inplasy2026.6.0056 Downloaded from https://inplasy.com/inplasy-2026-6-0056/

classifications, sex-related differences, and potential determinants of adverse prognosis. When available, data regarding pharmacological treatments, including contemporary heart failure therapies, and their association with clinical outcomes will also be collected and summarized. Furthermore, the review will identify existing knowledge gaps and areas requiring future mechanistic, prognostic, and therapeutic investigation.

Data management All records identified through the database searches will be screened for eligibility using a predefined study selection process. Titles and abstracts will first be evaluated, followed by full-text assessment of potentially relevant articles. Data extraction will be performed independently by two reviewers using a standardized data collection form developed specifically for this review. Extracted information will include study characteristics, participant demographics, clinical variables, laboratory findings, imaging and haemodynamic parameters, therapeutic data, and outcome measures. The extracted dataset will be cross-checked for accuracy and consistency, and any discrepancies will be resolved through discussion and consensus between reviewers. When necessary, original publications will be re-examined to verify the accuracy of the collected information. All study data will be stored in electronic spreadsheets and organized according to predefined variables to facilitate qualitative synthesis and quantitative analyses where appropriate.

Quality assessment / Risk of bias analysis The methodological quality and risk of bias of the included studies will be evaluated independently by two reviewers using the National Institutes of Health Quality Assessment Tool for Observational Cohort and Cross-Sectional Studies. This assessment will consider key domains such as clarity of the research question, definition of the study population, selection methods, exposure assessment, outcome measurement, follow-up adequacy, adjustment for confounding, and appropriateness of statistical analyses. Each study will be classified as having good, fair, or poor overall quality according to the number and relevance of fulfilled criteria. Any disagreement between reviewers will be resolved by discussion and consensus. The results of the quality assessment will be incorporated into the interpretation of the evidence, particularly when evaluating the robustness and reliability of clinical, imaging, haemodynamic, and prognostic findings.

Strategy of data synthesis A narrative synthesis will be undertaken to summarize and integrate the available evidence on heart failure with supranormal ejection fraction (HFsnEF). Study characteristics, patient demographics, clinical features, laboratory findings, imaging parameters, haemodynamic measurements, prognostic data, and therapeutic information will be systematically collected and summarized. Descriptive comparisons between HFsnEF and other heart failure phenotypes, particularly HFpEF, will be performed whenever sufficient data are available.
For continuous variables, pooled study-level descriptive estimates will be generated using weighted summary measures based on sample size. Clinical outcomes reported across studies will be synthesized qualitatively and, when appropriate, quantitatively. Random-effects meta-analytic models will be used to estimate pooled event rates for major clinical outcomes, including all-cause mortality and composite cardiovascular endpoints. Owing to expected variability in study populations, diagnostic criteria, follow-up duration, and outcome definitions, substantial clinical and methodological heterogeneity will be anticipated. Consequently, adjusted effect estimates will primarily be summarized narratively when quantitative pooling is not considered methodologically appropriate. Findings will be presented through tables, figures, and structured narrative summaries to provide a comprehensive overview of the available evidence.

Subgroup analysis Where sufficient data are available, subgroup analyses will be performed according to study characteristics and patient populations. Potential subgroup categories may include differences in the definition of supranormal ejection fraction, sex-specific versus non–sex-specific LVEF thresholds, acute versus chronic heart failure settings, hospitalized versus ambulatory populations, and geographic region. Additional subgroup analyses may explore variations according to age, sex distribution, study design, follow-up duration, and the presence of specific haemodynamic or imaging assessments, such as ventricular–arterial coupling or right ventricular–pulmonary arterial coupling evaluation. If an adequate number of studies report comparable outcome data, subgroup-specific estimates may be generated to explore potential sources of heterogeneity and to better characterize distinct clinical presentations of HFsnEF. However, all subgroup analyses will be considered exploratory and interpreted cautiously given the anticipated variability among the included studies.

Sonaglioni et al. INPLASY protocol 202660056. doi:10.37766/inplasy2026.6.0056 Downloaded from https://inplasy.com/inplasy-2026-6-0056/

Sensitivity analysis Sensitivity analyses will be conducted when sufficient data are available to evaluate the robustness of the main findings. These analyses may include the exclusion of studies with lower methodological quality, studies using substantially different HFsnEF definitions, studies with small sample sizes, or studies contributing disproportionately to heterogeneity. When quantitative pooling of event rates is performed, leave-one-out analyses may be used to assess the influence of individual studies on the overall estimates. Additional sensitivity analyses may explore the impact of different clinical settings, follow-up durations, and endpoint definitions. The results will be interpreted cautiously and used to identify potential sources of instability or heterogeneity in the evidence base.

Language restriction No language restrictions will be applied during study selection. Eligible articles published in any language will be considered for inclusion, provided that sufficient information can be obtained to assess eligibility.

Country(ies) involved Italy.

Keywords Heart failure; supranormal ejection fraction; HFsnEF; preserved ejection fraction; HFpEF; left ventricular ejection fraction; ventricular remodeling; ventriculo–arterial coupling; end-systolic elastance.

Dissemination plans The findings of this systematic review will be disseminated through publication in a peer-reviewed scientific journal and presentation at national and international cardiology and cardiovascular imaging conferences. The results are expected to contribute to the growing body of evidence regarding heart failure with supranormal ejection fraction (HFsnEF) and to support future research focused on its pathophysiology, prognostic significance, and therapeutic management. In addition, the review may serve as a reference for clinicians and investigators interested in refining the phenotypic characterization and risk stratification of patients across the preserved ejection fraction spectrum.

Contributions of each author

Author 1 - Andrea Sonaglioni - Author 1 drafted the manuscript.
Email: sonaglioniandrea@gmail.com
Author 2 - Giulio Francesco Gramaglia - The author contributed to the Search Strategy.
Email: giulio.gramaglia@unimi.it
Author 3 - Gian Luigi Nicolosi - The author revised the initial draft.
Email: gianluigi.nicolosi@gmail.com
Author 4 - Massimo Baravelli - The author read, provided feedback and approved the final manuscript.
Email: massimo.baravelli@multimedica.it
Author 5 - Michele Lombardo - The author read, provided feedback and approved the final manuscript.
Email: michele.lombardo@multimedica.it

Sonaglioni et al. INPLASY protocol 202660056. doi:10.37766/inplasy2026.6.0056 Downloaded from https://inplasy.com/inplasy-2026-6-0056/
